# Effect of Visuospatial Attention on the Sensorimotor Gating System

**DOI:** 10.3389/fnbeh.2019.00001

**Published:** 2019-01-15

**Authors:** Daisuke Ishii, Kotaro Takeda, Satoshi Yamamoto, Akira Noguchi, Kiyoshige Ishibashi, Kenya Tanamachi, Arito Yozu, Yutaka Kohno

**Affiliations:** ^1^Center for Medical Sciences, Ibaraki Prefectural University of Health Sciences, Ami, Japan; ^2^Department of Cognitive Behavioral Physiology, Chiba University Graduate School of Medicine, Chiba, Japan; ^3^Faculty of Rehabilitation, School of Health Sciences, Fujita Health University, Toyoake, Japan; ^4^Department of Physical Therapy, School of Healthcare, Ibaraki Prefectural University of Health Sciences, Ami, Japan; ^5^Sakai Neurosurgical Clinic, Hamamatsu, Japan; ^6^Department of Physical Therapy, Ibaraki Prefectural University of Health Sciences Hospital, Ami, Japan

**Keywords:** prepulse inhibition, sensorimotor gating, visuospatial attention, laterality, startle reflex, visual prepulse

## Abstract

The integration of multiple sensory modalities allows us to adapt to the environment of the outside world. It is widely known that visual stimuli interfere with the processing of auditory information, which is involved in the ability to pay attention. Additionally, visuospatial attention has the characteristic of laterality. It is unclear whether this laterality of visuospatial attention affects the processing of auditory stimuli. The sensorimotor gating system is a neurological process, which filters out unnecessary stimuli from environmental stimuli in the brain. Prepulse inhibition (PPI) is an operational measure of the sensorimotor gating system, which a weaker prestimulus (prepulse), such as a visual stimulus, inhibits the startle reflex elicited by a subsequent robust startling stimulus (pulse) such as a tone. Therefore, we investigated whether the visual stimulus from the left or right visual space affects the sensorimotor gating system in a “rest” task (low attentional condition) and a “selective attention” task (high attentional condition). In the selective attention task, we found that the target prepulse presented in the left and bilateral visual fields suppressed the startle reflex more than that presented in the right visual field. By contrast, there was no laterality of PPI in the no-target prepulse condition, and there was no laterality of PPI in the rest task. These results suggest that the laterality of visuospatial attention affects the sensorimotor gating system depending on the attentional condition. Moreover, the process of visual information processing may differ between the left and right brain.

## Introduction

We recognize the environment of the outside world through multiple sensory modalities, such as the visual and auditory modalities. Adaptation to the environment is achieved by integrating these multiple sensory modalities. It has been shown that the processing of visual and auditory information interfere with each other, which is involved in the ability to pay attention (Richard et al., 1988; Pomper and Chait, 2017).

The right hemisphere plays an important role in visuospatial and auditory attention (Kinsbourne, 1977; Corbetta et al., 1993; Thiebaut de Schotten et al., 2011; Duecker and Sack, 2015). In the visuospatial attention, the hemispatial neglect is most common in damage to the right cerebral hemisphere, which causes visual neglect of the left visual space (Stone et al., 1992). These studies have indicated that the visuospatial attention exhibits laterality.

The sensorimotor gating system is a neurological process that filters out unnecessary stimuli from environmental stimuli in the brain. Prepulse inhibition (PPI) of the startle reflex is an operational measure of the sensorimotor gating system. PPI reduces the amplitude of the startle reflex that occurs when a prepulse (visual, auditory, or tactile stimulus) is presented prior to the startling stimulus (Graham, 1975; Blumenthal and Gescheider, 1987; Luthy et al., 2003). The attention to a prepulse or negative emotional experience has been shown to enhance PPI (Luthy et al., 2003; Ishii et al., 2010), suggesting that the sensorimotor gating system changes with a variety of internal and external conditions. It is unclear whether this laterality of the visuospatial attention affects the sensorimotor gating system.

Given this background, we investigated whether visual stimuli from the left or right visual space affect the sensorimotor gating system using two tasks, a “rest” task (low attentional condition) and a “selective attention” task (high attentional condition). In the future, it will be important to investigate the effects of visuospatial-attention laterality on the processing of other sensory modalities, in order to elucidate the pathological mechanisms of attention-related illnesses such as hemispatial neglect or attention disorders, which make it difficult to properly process information from the environment.

## Methods

### Subjects

Ten healthy right-handed male subjects (mean age: 28.9 years, range: 20–40 years) participated in this study after giving informed consent. None of the participants had a history of neurological or psychiatric disease or any condition associated with auditory and visual system abnormalities, as determined by a non-structured interview. All subjects were able to understand the instructions and gave written informed consent. Subjects were excluded if they did not satisfy the following criterion: the average integrated electromyogram (iEMG; startle reflex) within 40–140 ms from the onset of startle stimuli in each prepulse-pulse condition was less than the mean + 3 standard deviations of the baseline (iEMG for 100 ms just before the onset). The study was performed in accordance with the Declaration of Helsinki. Approval of the protocol was obtained from the ethics committee of the Ibaraki Prefectural University of Health Sciences (approval number: 800).

### Experimental Design

All participants underwent two experimental tasks (rest task and selective attention task) in random order; these were counterbalanced across subjects on two separate days with an interval of at least three days between the tasks. Subjects were asked about their smoking on the experimental day as well as their sleeping hours the night before the experiment. Moreover, before the session, alertness was assessed using the Stanford Sleepiness Scale (Hoddes et al., 1973; Rumpf et al., 2017).

### Apparatus and Stimuli

**FIGURE 1:**
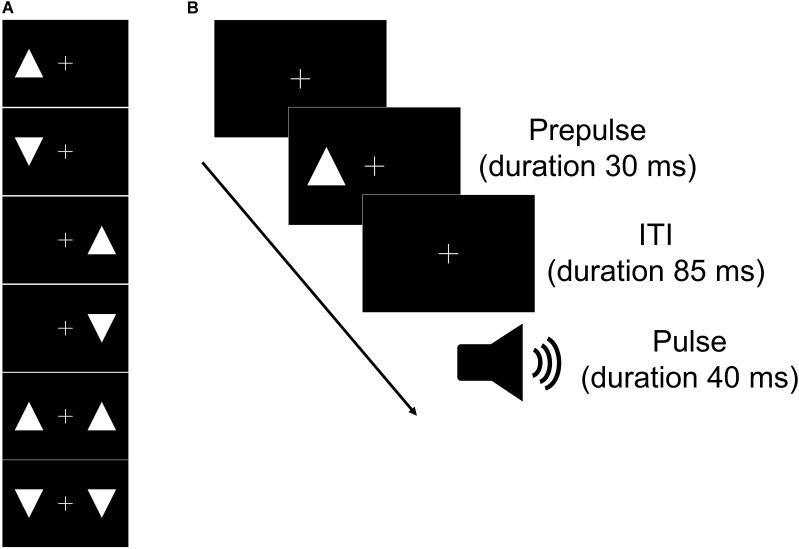
Schematic of the types of prepulse **(A)** and the time course of one trial of the task **(B)**. **(A)** Six visual stimuli were used in the experiment as prepulses. Upward and/or downward triangles were presented on the left and/or right across a fixation cross for the visual stimuli. **(B)** The prepulse (visual stimulus) could appear following the fixation cross. The pulse (tone) was presented at 115 ms after the prepulse presentation. ITI, interstimulus interval.

Acoustic stimuli were administered binaurally through headphones (ATH-PRO5MK3; Audio-Technica, Tokyo, Japan). We used two acoustic stimuli: a tone burst stimulus (white noise, 110 dB SPL; duration = 40 ms; rise/fall time = 5 ms) as a pulse to elicit a startle reflex and continuous background white noise (68 dB). Visual stimuli were presented on an LCD monitor with a 60 Hz refresh rate (XU2390HS; MouseComputer, Tokyo, Japan) at a viewing distance of 100 cm. All stimuli appeared white against a uniformly black background. We used six visual stimuli as prepulses (Figure 1A). All prepulses appeared at symmetrical locations in the left and right visual fields at the same height as the fixation cross. All stimuli were randomly presented across the experiments using PsychToolBox for Matlab R2017b (MathWorks, Natick, MA, United States).

### Rest Task

The rest task began with a 2-min acclimation period consisting of 68 dB continuous white noise via headphones. After the acclimation period, the subjects received one tone burst stimulus. The initial tone burst stimulus was followed by 7 trials in six blocks (a total of 42 trials). Each block had a pulse alone trial and six visual prepulse-pulse trials in which a prepulse preceded the pulse at 85-ms intervals ordered pseudo-randomly. The sequence of events for prepulse-pulse trials is shown in Figure 1B. After the 43 trials, the subjects received one tone burst stimulus. During the task, the subjects were instructed to relax and maintain their gaze on the central cross. The session lasted approximately 12 min (interstimulus interval = 9–22 s).

### Selective Attention Task

In the selective attention task, the apparatus and stimuli were identical to those of the rest task. The subjects were instructed to silently count the number of occurrences of a target prepulse (white upward triangle or white downward triangle) while maintaining their gaze on the central cross. The target prepulse was randomly selected for each participant.

### Psychophysiological Data Collection and Analysis

Disposable gelled EMG electrodes (Mets, Tokyo, Japan) were placed on the left orbicularis oculi muscle. The ground electrode was attached to the forehead. EMG data were collected at a sampling frequency of 10 kHz with custom-made LabView software (National Instruments Japan, Tokyo, Japan) from –100 to 200 ms of the onset of the pulse stimulus. A bandpass filter was set at 28 Hz to 500 kHz. A notch filter was also applied to eliminate the 50 Hz line noise. The EMG signals were rectified, integrated with a time constant of 10 ms (iEMG), and then averaged for each condition. The first eight and the last trials were excluded from the average. When the excessive activity due to eye-blinking overlapped with baseline period or startle reflex phase, these trials were also excluded from the average. The peak in the averaged iEMG within 40–140 ms from the onset of the startle stimuli was determined as the maximal amplitude for the startle reflex. All the signal processing was performed using MATLAB R2017b.

### Statistical Analysis

%PPI was calculated as {[(Peak of the averaged iEMG in pulse-alone trial) – (Peak of the averaged iEMG in prepulse-pulse trial)]/Peak of the averaged iEMG in pulse-alone trial}× 100. We performed a one-way repeated-measures analysis of variance (ANOVA) on the %PPI to compare among the prepulse conditions for each task. Bonferroni’s correction was used for *post hoc* comparisons when ANOVA revealed statistically significant differences. The level of statistical significance was set at *p* < 0.05. All the analyses were performed using SPSS Statistics 23.0 for Mac (IBM, Armonk, NY, United States).

## Results

### Pre-task Vigilance Levels and Smoking Status

In the rest task, the average total sleeping time on the day before the experiment was 6.55 h (SD 0.96). The median Stanford sleepiness scale was 2 (interquartile range, 1.8 to 3.0). In the selective attention task, the sleeping time was 6.25 h (1.32). The median Stanford sleepiness scale was 2 (1.0 to 3.0). There was no significant difference of the sleeping time between the experimental tasks (paired *t*-test). Three subjects smoked on each experimental day.

### %PPI in the Rest Task

**FIGURE 2:**
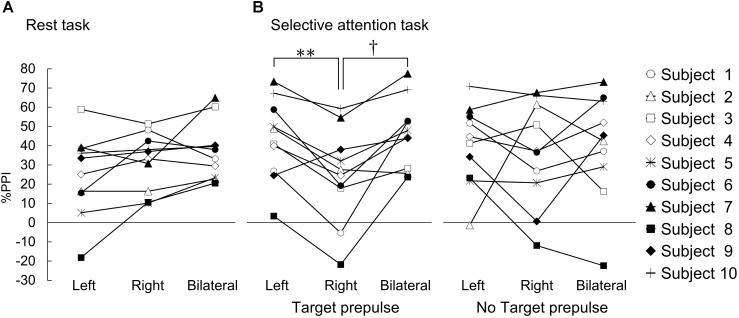
%PPI in the rest task and the selective attention task. The results for each individual subject are shown. %PPI in the rest task **(A)** and the selective attention task **(B)**. **(A)** %PPI in each prepulse (left, prepulse presented on the left visual field; right, prepulse presented on the right visual field; bilateral, prepulse presented on both visual fields). **(B)** %PPI under the target prepulse (left panel) and no-target prepulse (right panel) conditions. ^∗∗^, ^†^Bsonferroni-corrected *p* < 0.01 and *p* < 0.05, respectively.

One-way repeated measures ANOVA revealed a significant main effect of the prepulse conditions (*F*(2, 18) = 4.377, *p* < 0.05, η^2^_p_= 0.33) (Figure 2A). By contrast, a *post hoc* comparison indicated that there was no difference in %PPI in the rest task among the different prepulse conditions (Bonferroni corrected *p* > 0.05; left vs. right, *d* = 0.37; left vs. bilateral, *d* = 0.66; right vs. bilateral, *d* = 0.36).

### %PPI in the Selective Attention Task

In the responses to the target prepulse, one-way ANOVA revealed a significant main effect of the prepulse conditions (*F*(2, 18) = 10.645, *p* < 0.01, η^2^_p_ = 0.54) (Figure 2B). A *post hoc* comparison indicated that the %PPI at the target prepulse presented in the right visual space was significantly lower than the %PPIs for the target prepulses presented in the left and bilateral visual spaces (right vs. left Bonferroni corrected *p* < 0.01, *d* = 0.82; right vs. bilateral, Bonferroni corrected *p* < 0.05, *d* = 1.02). On the other hand, there was no difference between the left and bilateral visual spaces (left vs. bilateral, *d* = 0.16).

In the responses to the “no-target” prepulse, the one-way ANOVA showed no significant main effect of the prepulse conditions (*F*(2, 18) = 0.204, η^2^_p_ = 0.02) (Figure 2B).

### Percentages of Correct Answers in the Selective Attention Task

The average correct answer rate of the selective attention task was 91.7% (SD 10.2; range 72.2–100%).

## Discussion

This study revealed that the lower %PPI could be shown only in the prepulse presented in the right visual field in the selective attention task. This result suggests that visual stimuli from the left and right visual fields have different effects on the sensorimotor gating system under the high attentional condition. Additionally, there was no laterality of the interference effect of the prepulse on the sensorimotor gating system when attention was not directed to the prepulse or the subjects were asked to ignore the prepulse.

It is known that paying attention to a prepulse enhances PPI (Luthy et al., 2003). Additionally, healthy humans show a leftward bias in visuospatial tasks (Jewell and McCourt, 2000). For the visuospatial attention, the right hemisphere plays an important role, and the attention exhibits right hemisphere dominance (Kinsbourne, 1977; Corbetta et al., 1993; Thiebaut de Schotten et al., 2011). The current study showed that there was no laterality of PPI under the lower attentional conditions. Our study and previous studies suggest that the laterality of the visuospatial attention is not always present.

In the pointing reaction to the visual target, the reaction time for the left hand has been reported to be shorter than that for the right hand, suggesting that the processing of visual information related to the spatial parameterization of the movement is faster in the right hemisphere than in the left hemisphere (Carson et al., 1990). In our study, the higher %PPI could be seen in the prepulse presented in the left visual field in the selective attention task. Our results and this previous study suggest that the processing of visual information in the right brain may be faster than that in the left brain under the high attentional condition and have a great effect on subsequent reaction.

The transcallosal fibers that connect the two hemispheres of the cerebral cortex mediate interhemispheric inhibition (Asanuma and Okuda, 1962; Ferbert et al., 1992). The right human posterior parietal cortex associated with the visuospatial attention exerts strong inhibitory activity over the contralateral homologous area (Heilman and Valenstein, 1972; Hilgetag et al., 2001; Cazzoli et al., 2009; Koch et al., 2011). We speculate that the right hemisphere, with increased excitability, under the high attentional condition may inhibit the activity of the left hemisphere through the transcallosal fibers, which may induce laterality of the visuospatial attention.

A previous study revealed that PPI can be enhanced by smoking (Kumari et al., 1996). This effect is canceled by short-term abstinence from smoking (Della Casa et al., 1998). In our study, smokers had abstained from smoking for 3 h before the experiments. Additionally, the data of smokers were not obviously different from that of non-smokers in the experiments. There was also no significant difference in the sleeping status between the experiments. These results suggested that the laterality of the PPI was not due to a change in smoking status or vigilance.

We showed that the interference effects of visual stimuli on the auditory gating system changed dynamically depending on the attentional conditions. Our study might shed light on the pathology of attention-related illnesses such as hemispatial neglect or attention disorders.

## Author Contributions

DI, KoT, SY, AN, and YK conceived of the presented idea and designed the experiments. DI, SY, and AN carried out the experiments. DI, KoT, and KeT analyzed the data. DI, KI, AY, and YK interpreted the results. DI and KoT drafted the manuscript.

## Conflict of Interest Statement

The authors declare that the research was conducted in the absence of any commercial or financial relationships that could be construed as a potential conflict of interest.
